# Supplementary material for: How do multimedia and blended learning enhance music elective courses? examining the roles of learning attitudes, styles, and teaching presence
Source: PLoS One. 2025 Jun 25;20(6):e0326037. doi: 10.1371/journal.pone.0326037 (PMC12192290; doi:10.1371/journal.pone.0326037)
Supplement: S1 Appendix — (PDF) [file pone.0326037.s001.pdf]

Appendix I Questionnaire

Survey Questionnaire for College Music Elective Courses

Dear classmate:

Thank you for participating in this survey and research on college music elective courses. This questionnaire aims to understand your views and satisfaction with various aspects of music elective courses in universities. Your answer will help us with the methods and approaches of music elective education to better serve students. Please fill out the questionnaire based on your true situation and feelings. All answers will be strictly confidential and will only be used for academic research.

Part1 Basic Information

How old are you?

18-20 years old

21-23 years old

24-26 years old

What is your gender?

male

female

What is your major?

Philosophy, economics, law, education, literature, history, science, engineering, agriculture, medicine, military science, management, art

What is your grade level?

Freshman

Sophomore

Junior

Senior

Part 2 Main part (please mark a "√" in the corresponding column based on your situation)

Note: please, indicate your opinions about each of the questions below by ticking one of the five responses provided in the column on the right side. The response scale ranges from "Strongly disagree" to "Strongly agree". You may choose any of the five possible responses, as each represents a degree on the continuum. 5 4 3 2 1 SA A N D SD

Construct	Items	5<br>SA	4<br>A	3<br>N	2<br>D	1<br>SD
**Perceived playfulness**	1. I think the music course content is lively and interesting.					
	2. Activities in music class can enhance my interest in learning.					
	3. Music courses will stimulate my curiosity.					
	4. I feel very happy during music class.					
**Cognitive presence**	1. The music questions raised by the teacher may arouse my interest in learning.					
	2. Brainstorming and searching for relevant information can help me solve problems related to music course content.					
	3. Course discussions help me understand different perspectives and viewpoints.					
	4. Reflecting on the course content and discussions can help me understand the fundamental content of this music course.					
	5. In music courses, I can effectively apply theoretical knowledge to practice.					
**Teaching presence**	1. The instructor clearly conveyed the course objectives.					
	2. The teaching staff provided clear guidance on how to participate in learning activities.					
	3. The instructor helps students complete their learning tasks by answering questions and providing guidance.					
	4. The instructor provided constructive feedback in the music course.					
	5. The teaching staff helps students effectively participate in classroom discussions.					
**Social presence**	1. In music classes, I feel that there is good interaction with other classmates.					
	2. I can easily share and exchange ideas with my classmates in music classes.					
	3. The group activities in the music course helped me better understand the course content.					
	4. I think interacting with classmates in music classes is					

	enjoyable and helpful.					
	5. In music classes, I feel like I am a part of the class community.					
**Perceive course quality**	1. I believe that the content of music courses is of high quality.					
	2. The teaching materials and resources for music courses are very helpful.					
	3. The arrangement and structure of music courses are reasonable and effective.					
	4. The teaching methods of music courses can meet my learning needs.					
	5. Music courses provide sufficient practical opportunities to apply the knowledge learned.					
	6. Music courses can effectively enhance my music skills and knowledge.					
**Perceived usefulness**	1. I believe that the content of music courses is helpful for my learning and development.					
	2. I can apply the knowledge and skills learned from music courses in my daily life.					
	3. The music course has improved my understanding and appreciation of music.					
	4. Music courses have boosted my confidence.					
**Satisfaction**	1. I am overall satisfied with the music course.					
	2. The music course met my learning expectations.					
	3. I am satisfied with the teaching methods of music courses.					
	4. The content of music courses is very helpful for my learning.					
**Learning attitude**	1. I am full of enthusiasm for learning music courses.					
	2. I maintain a high level of learning motivation in music courses.					
	3. I am willing to invest more time and energy in music courses.					
	4. I am willing to accept new challenges in music courses.					
**Learning style**	1. I like to learn music by listening and repeatedly imitating.					
	2. I learn music courses by watching videos and demonstrations.					
	3. I achieve better learning outcomes through group collaboration and discussions.					
	4. I enjoy mastering music skills through independent learning.					
**Continuous**	1. I plan to continue taking more music courses.					
	2. I am interested in exploring more music related courses.					

**learning intention**	3. I would recommend other students to also take music courses.					
	4. Even when faced with other course choices, I will still prioritize elective music courses.					
	5. I believe that continuing to study music courses will be beneficial for my overall development.					

Appendix II Music Class

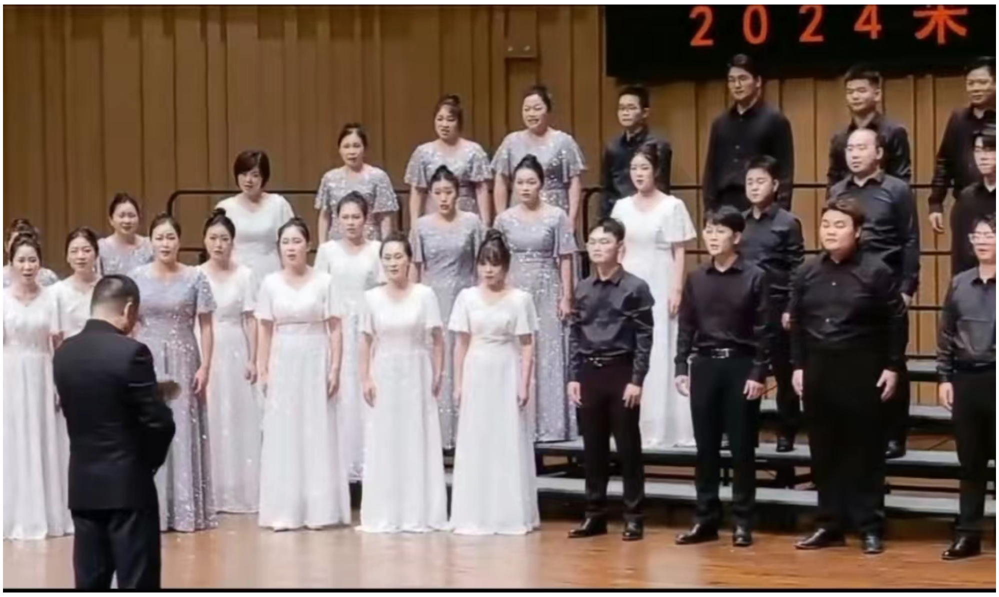

Figure 1: Music Class—Classmates pretend to be very formal and prepare to present their performance in music class (Source: Photo taken by the author during class)

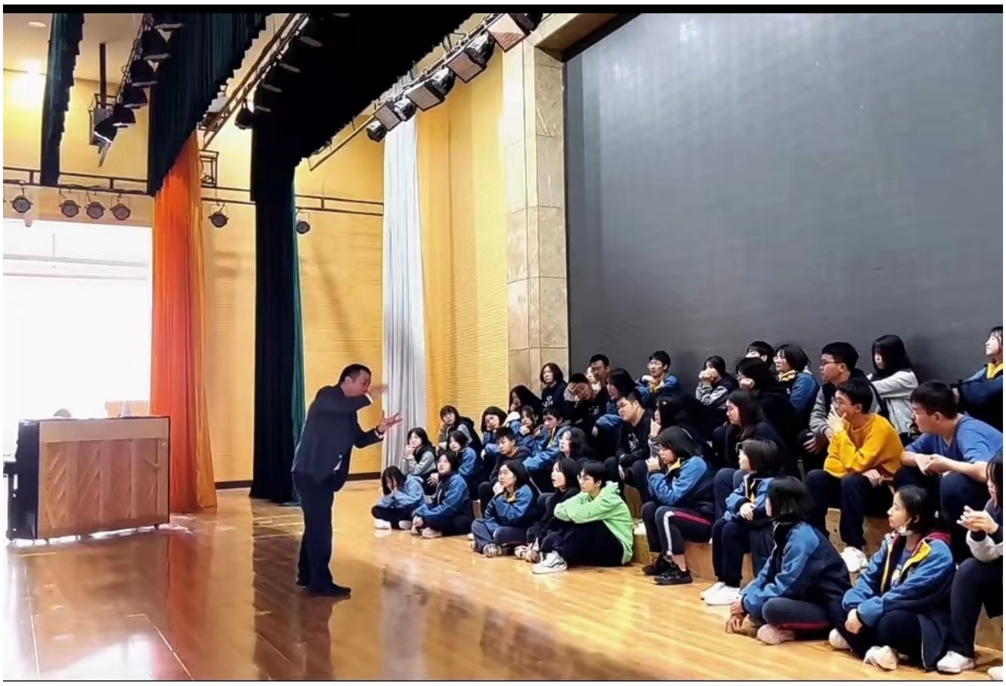

Figure 2: Music Class—Classmates are dressed casually and sitting on the ground listening to the teacher's explanation (Source: taken by the author during class)
