# Supplementary material for: How do multimedia and blended learning enhance music elective courses? examining the roles of learning attitudes, styles, and teaching presence
Source: PLoS One. 2025 Jun 25;20(6):e0326037. doi: 10.1371/journal.pone.0326037 (PMC12192290; doi:10.1371/journal.pone.0326037)
Supplement: Attachment — Submitted filename: PONE-D-24-33225 Reviewer Recommendation.pdf [file pone.0326037.s003.pdf]

This study provides valuable insights into the effects of multimedia and blended learning within music elective courses. Although this study contributes meaningfully to both theoretical and practical fields, I suggest that author strengthen the academic rigor and relevance of your paper by elaborating on and refining several critical elements.

1. Introduction

1) The lines 6-7 of the first paragraph mentions, "According to the guidelines of the Chinese Ministry of Education for aesthetic education in higher education institutions.". It is essential to provide the publication year, thereby facilitating readers in locating and verifying the source of this information.
2) The lines 8-9 of the first paragraph mentions, "music elective courses in universities have always attracted numerous students with their unique charm.". Are there particular data sets or research studies that support this assertion? For example, what proportion of students participates in music courses, and how do these figures contrast with those of other elective offerings?
3) The last sentence of the first paragraph mentions, "in the fast-paced modern society, to stimulate students' sustained interest in music elective courses has become an interesting research context that is worth further investigation.". This could be elaborated by discussing why this is an interesting research context, and how such sustained interest could potentially impact students' long-term learning outcomes and psychological well-being.
4) The line 1 of the second paragraph mentions, "learning intention (LI) has been studied extensively", however, it does not provide a clear definition of the term or elucidate its significance within the specified context. The authors are encouraged to include more detailed definitions or contextual information within the manuscript.
5) The line 3-5 of the second paragraph mentions, "Music elective course teaching has been innovated in recent years by Internet and teaching methods to effectively meet the needs of students' physical and mental development." However, the manuscript does not provide specific information regarding the mechanisms through which these innovations have been enacted. For instance, it remains unclear whether these advancements are supported by an online platform, or specific interactive technologies. Additionally, while the manuscript asserts that "effectively meet the needs of students' physical and mental development.". But, it would be advantageous to include concrete examples or evidence that demonstrate the effectiveness of these new approaches in meeting those needs. For instance, is there empirical data available concerning student feedback, improved learning outcomes, or enhancements in mental health?
6) The second paragraph mentions, the manuscript transitions directly from discussing learning intentions to statements about innovating music elective courses through the internet and teaching methods, without a bridge linking these two concepts. There is a lack of explanation on how the internet and new teaching methods respond to or promote the development of learning intentions.

It is recommended that the author consider adding some explanations or data to support this connection.

7) The line 5-7 of the second paragraph mentions, “Even though multiple studies have shown that specific elective courses can improve students' performance in each subject area, and some studies have also evaluated student satisfaction at the end of the course, ”, please cite the relevant literature.
8) The line 7-9 of the second paragraph mentions, “there has not been a study conducted to determine students' expectations and continuous learning intention before taking music elective courses.”. Nevertheless, this assertion requires substantiation through a comprehensive review of the existing literature to establish the existence of this research gap.
9) The line 9-10 of the second paragraph mentions, “The preference of college students for the teaching methods of music elective courses has not been evaluated.”. Please cite the relevant literature.
10) The line 5-6 of the third paragraph mentions, “Maximizing teaching effectiveness via the integration and optimization of different teaching resources.”. This statement necessitates a grammatical adjustment.
11) The line 8-15 of the third paragraph mentions, the text addresses both practical perspective and theoretical perspective， however, it does not provide explicit methodologies for their integration. For example, the text does not clarify how to effectively merge the teaching of traditional music theory with practical performance. The incorporation of examples or comprehensive descriptions of specific pedagogical strategies would significantly improve the clarity and applicability of the discourse.
12) The last sentence of the third paragraph mentions, “This study further promotes the formation of a detailed mechanism for the relationship between the above factors.”. This formulation is relatively abstract. It is advisable to include specific information concerning the methodologies utilized to investigate or substantiate these mechanisms.

2. Theoretical background

13) The literature review section primarily highlights positive outcomes. Has there been any deliberation regarding the inclusion of opposing viewpoints or negative instances to augment the thoroughness and depth of the manuscript?

2.1

14) The lines 2 of the first paragraph mentions, Bhattacharjee’s concept is mentioned，please cite the relevant literature.
15) The lines 5 of the first paragraph mentions, the text experiences a sudden shift from theoretical to information systems. To improve the coherence of the discourse, it would be advantageous to incorporate one or two transitional sentences.
16) Literature cited in manuscript by Ng, Davy TK and Yao et al.

2.2

17) The first paragraph mentions,the TAM and its two fundamental components, but the ECM-ISC is suddenly introduced later without a clear explanation of the

relationship between these two models.This deficiency in clarity may result in confusion for the reader, as the justification for the introduction of the ECM-ISC model, along with the referenced literature, is not clearly articulated. It is advisable to include transitional sentences to improve the logical coherence between the two models.

3. Hypotheses

3.1

18) The term "Learning attitude" is defined at the beginning of the paragraph, but subsequently, it is used interchangeably with "Learning motivation", which may lead to conceptual confusion. It is recommended to clearly differentiate these two concepts or explicitly clarify their relationship.
19) Could author supply further academic literature that specifically examines the correlation between "Learning attitude" and "continuous learning intention", in order to strengthen the scholarly foundation for the hypothesis?

3.3

20) Hypothesis H3 "Student satisfaction positively correlates with learning attitude.". The preceding discourse predominantly highlights the connection between learning satisfaction and continued learning. It would be prudent to incorporate a discussion regarding the impact of satisfaction on attitudes toward learning before articulating the hypothesis.

3.4

21) Line 8: citation format needs to be standardized

3.5

22) "Perceive quality" should be corrected to "Perceived quality" throughout the document to maintain consistency and accuracy.

3.6

23) The line 14 of the paragraph mentions, "Hu confirmed that social presence is an important predictor of online learner satisfaction.". Please cite the relevant literature.

3.7

24) The text defines "Teaching presence refers to students' perception of the existence of teachers.". However, this definition is somewhat succinct and may prompt readers to inquire whether it specifically addresses the physical presence of the teacher or the teacher's engagement and participation in the educational process. It is advisable to provide a more detailed and nuanced definition.
25) Suppose there is a small error in H8: an extra "e".

3.9

26) Please cite the relevant literature for the first sentence

4. Experimental results

4.1

27) The paragraph does not mention how the data were collected or the representativeness of the sample. Additionally, it should be clarified which methods were used for data analysis.

4.2

28) Ensure that all textual statements are directly supported by the data presented in the figures. As shown in the figure, students aged 18-20 have a higher proportion of choosing “agree,” “do not care,” “strongly disagree,” and “disagree.” Students aged 21-23 are more likely to choose “strongly agree.” Female students have higher proportions of choosing “strongly agree,” “do not care,” and “strongly disagree” compared to male students. The highest proportion of choosing “agree” is among juniors and freshmen, while the highest proportion of choosing “strongly agree” is among juniors and seniors.

4.3

29) If possible, please provide the findings of the Bartlett test.

4.4

30) The text indicates that the GFI is 0.877 < 0.9. This presents a logical inconsistency, as 0.877 is less than 0.9.

4.5

31) For the hypotheses that were not supported, could the author provide explanations or theoretical discussions? This is crucial for understanding the complexity and depth of the research findings.
32) There is no table 9 and the serial numbers need to be changed.

5. Implications

5.2

33) The line 4 of the paragraph paying attention to the correct use of punctuation.

6. Conclusion

34) What particular recommendations does the research offer regarding the design and execution of music elective courses in practice? Furthermore, can practical pedagogical strategies be suggested based on the study’s findings?
35) The study doesn’t adequately discuss the limitations of the study, particularly regarding the representativeness of the sample in relation to the wider student population in China. Furthermore, it is essential to evaluate whether the data collection and analysis methods utilized may introduce potential biases.
36) Kindly adjust the manuscript format to conform to the specifications outlined by PLOS ONE.
