# Supplementary material for: How do multimedia and blended learning enhance music elective courses? examining the roles of learning attitudes, styles, and teaching presence
Source: PLoS One. 2025 Jun 25;20(6):e0326037. doi: 10.1371/journal.pone.0326037 (PMC12192290; doi:10.1371/journal.pone.0326037)
Supplement: Attachment — Submitted filename: Rebuttal_Letter_3_Nov 2.pdf [file pone.0326037.s005.pdf]

Dear Editor,

We sincerely appreciate the reviewers' comments and suggestions as well as feel encouraged by their constructive comments. We are submitting the revised manuscript together with this "Rebuttal letter" for your consideration.

The changes have incorporated the suggestions of the reviewers, and we thank the reviewers for their time taken to read the manuscript and provide us with helpful and constructive comments. All the queries/comments of the reviewers have been addressed as below. All the authors have checked the manuscript and agreed to the submission.

Warm regards,

Pingbo Tang
Hunan Vocational College of Science and Technology

3, Nov, 2024

Reviewer #1:
This study aims to identify interactions and causalities between the factors regarding e-learning in music elective courses. I think this study has shortcomings. My recommendations are listed below.
1. Remove the title “3.10. Conceptual Model.” Instead of this, a short explanation of the conceptual model should be given after the title “3. Hypothesis." And Figure 1 should be given in this part.

Thanks for your careful review and valuable feedback. Regarding your suggestion to move the title and content of "3.10. Conceptual Model" to follow the "3. Hypothesis" section, provide a brief explanation of the conceptual model, and place Figure 1 in that section. We understand that you aim to simplify the structure and strengthen the logical connections between sections.

3. Hypotheses

3.1~~0.~~ Conceptual model

The conceptual model is shown as Fig. 2~~:–~~, examines the relationships between various factors influencing students' continuous learning intention. Key constructs include perceived playfulness, cognitive presence, teaching presence, social presence, and perceived quality, all of which impact perceived usefulness and satisfaction. These in turn affect learning attitude, learning style, and ultimately, continuous learning intention. This model provides a comprehensive framework for testing hypotheses about how these constructs interact within a blended learning environment in music education.

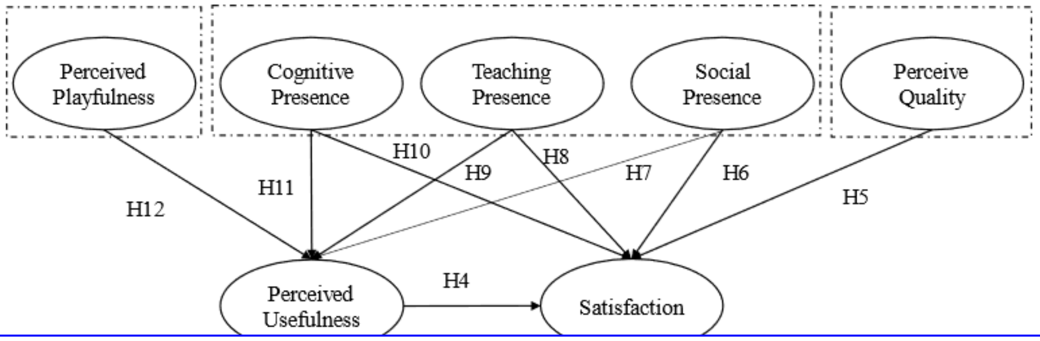

2. The literature shows that the factor "trust" was integrated with TAM. Why didn’t you examine the trust factor's relationship and influence?

1) Thank you for your valuable comments and for highlighting the "trust" factor in the Technology Acceptance Model (TAM). We recognize that trust is crucial in many TAM applications, particularly in areas emphasizing technology reliability and security.

2) Our study, however, focuses on the educational impact of multimedia and blended learning in music elective courses. For this reason, we prioritized factors such as

perceived usefulness, satisfaction, and learning motivation, which directly influence student engagement and continuous learning intention. Integrating trust as a primary variable might divert focus from these educationally relevant factors.

3) To address your point, we add a discussion on the potential role of trust in our revised manuscript, acknowledging its impact on perceptions in educational contexts. We believe this addition will enrich our theoretical framework without altering the main focus of our study.

Thank you again for your insightful suggestions.

individuals judging whether this technology is beneficial to themselves through their own knowledge, experience, and recommendations from others; (2) individuals evaluate their satisfaction with a new technology based on their existing knowledge and skills (Adileh, 2012). The focus of this model is to understand how users accept and use new technologies. This is especially critical in educational environments for improving learning efficiency. To further explore the dynamic process of technology acceptance, the information system continuous use model (ECM-ISC) was introduced. The information system continuous use model (ECM-ISC) reconstructed by Bhattachaijee includes four variables: expected confirmation, satisfaction, intention to continue using, and perceived usefulness. It emphasizes the relationship between expectation confirmation and satisfaction in the process of technology use. Specifically, expectation confirmation refers to users' expectations of the functionality and effectiveness provided by the technology, while satisfaction is the evaluation of these expectations after actual use. This further influences users' intention to continue using the technology, thereby complementing the core components of the TAM model. Perceived usefulness and satisfaction were included as important predictive factors and played a mediating role in this study. It shows that while TAM and ECM-ISC are two

3.The population of research is not given in the study. If you adopted the sampling, how did you select the sample? What is the sampling method? What is the size of the sample? If you targeted a limited population, what are the characteristics of this target population? And what is the response rate of participants? The answers you give show us the power and validity of your research.

1) First, regarding the research subjects, our survey was conducted at Hunan University of Science and Technology, focusing primarily on university students from different grades. To ensure the representativeness of the sample, we used a stratified sampling method, aiming to cover students from the first to the fourth year. Specifically, we distributed a total of 400 questionnaires, with 200 distributed online and the other 200 distributed offline. Ultimately, we received 315 valid questionnaires, resulting in a valid response rate of 78.75%. This indicates that our sample size is relatively sufficient,

contributing to the robustness of the research results.

2) Second, regarding the sample characteristics, participants come from different grades, which reflects the variations in learning experiences and academic achievements among students of different years. Additionally, during the data collection process, we ensured that all questionnaires were conducted anonymously to protect students' privacy and ensure the authenticity of the data. During the questionnaire filling process, we provided students with sufficient time and space to thoughtfully consider each question, further enhancing the validity and reliability of the questionnaires.

ensure the measurement tool more scientific and reliable. ~~The detailed measurement items are shown in Table 1, and the reliability and validity of the questionnaire are ensured through extensive surveys and data analysis. The survey was conducted from January 12, 2024 to May 29, 2024, and a total of 315 valid questionnaires were collected, at Hunan Vocational College of Science and Technology, and the ethics committee waived the need for consent.~~ This survey was conducted from January 12, 2024, to May 29, 2024, at Hunan University of Science and Technology. A stratified sampling method was used to target first-year, second-year, third-year, and fourth-year students at the university. We distributed 400 questionnaires. Half of these (200) were distributed online, while the other half (200) were distributed offline. In the end, we collected 315 valid questionnaires. Data collection was done anonymously to ensure students' privacy and the authenticity of the data. When collecting data, we also ensured that students had enough time and space to carefully think about each question. This helped improve the validity and reliability of the questionnaire.

4. I noticed you applied SEM in your research. But you did not employ the Confirmatory Factor Analysis (i.e. the Measurement Model), and the exploratory factor analysis was used. Why did you employ this approach?

We explain our research rationale and method selection as follows:

Initially, our study aimed to explore the relationship between students' learning experiences and academic achievements. To achieve this, we developed a preliminary measurement tool and employed EFA to identify potential factor structures and assess the aggregation and independence of each item. EFA revealed underlying data patterns, laying the foundation for subsequent model development.

Additionally, EFA helped identify key constructs aligned with our research objectives, supporting the subsequent CFA. Once the factor structure matched our theoretical framework, we conducted SEM to verify the robustness and validity of these relationships. Following this, CFA was used to assess the measurement model fit and the interrelationships among constructs, confirming the theoretical and empirical validity of the factors and enhancing data performance across dimensions. Although we initially used EFA, we conducted comprehensive CFA in the later stages to ensure the

measurement tool's reliability and validity.

5. "This study explored the potential factor structure in measurement tools using principal component analysis (PCA) and variance maximization rotation methods." You should give reasons why you selected these methods. For example, the maximum likelihood method and the oblique rotation method can be used.
6. As I understand it, you used Eigenvalue 1 to determine the factors. Why did you employ this approach?

We appreciate the opportunity to clarify our methodological choices, specifically the use of PCA, rotation methods, and the eigenvalue >1 criterion for factor determination.

Choice of PCA: We selected PCA as our primary analytical method because it is a robust technique for data reduction and structural detection, ideal for exploring the factor structure of our measurement tool. PCA effectively identifies underlying patterns and extracts principal components that account for the majority of the variance, simplifying the data structure and reducing analytical complexity. Its stability and interpretability, especially with high-dimensional data, made it more suitable for our goal of uncovering latent variable structures compared to other methods like Maximum Likelihood (ML) or oblique rotation.

Eigenvalue >1 Criterion: We employed the eigenvalue >1 criterion, based on the Kaiser standard, to determine the number of factors. This widely accepted method ensures that only factors explaining a significant amount of variance are retained, as factors with eigenvalues below 1 contribute minimally to the data structure.

Cumulative Variance Consideration: Beyond the eigenvalue criterion, we also assessed cumulative variance. The first five principal components accounted for 52.873% of the total variance, demonstrating their effectiveness in explaining the measurement tool's variability.

Thank you again for your insightful feedback.

7. "Second, according to indicators such as NFI, RFI, IFI, TLI, and CFI, all exceeded the standard value of 0.9." As evidenced by this phrase, you stated that all goodness of fit test results for your model ensure the standards. However, when I looked at Table 4, I noticed that NFI, GFI and RFI were 0.87. These values did not meet the requirements for goodness of fit.
8. You cite research that include judgment standards for goodness of fit tests and reliability and validity analyses.

9. The term "correlation" in your hypothesis is not correct because you applied SEM in your study.
10. Add a discussion section before the implications. The results obtained in this study should be discussed in depth with the studies on the subject in the literature.

Thank you for your thorough review and valuable feedback. We acknowledge that the NFI, RFI, and GFI values did not reach the 0.9 standard. However, values close to 0.9 can still indicate a good fit, especially as they are common in research. We used multiple fit indices in our analysis, and although the NFI, RFI, and GFI were 0.877, 0.867, and 0.877, respectively, other indices such as CMIN/DF, IFI, TLI, and CFI all exceeded 0.9, supporting the validity of our model.

Regarding reliability and validity, our study reported a Cronbach's alpha of 0.929, indicating high internal consistency, and a KMO value of 0.901, which indicated that the sample was suitable for factor analysis. Thank you again for your constructive comments.

style has a negative impact on learning attitude. In terms of interaction, the interaction term between student satisfaction and learning style shows a significant moderating effect. The estimated coefficient of interaction effect is 0.2449, with a standard error of 0.0401, and the ~~correlation~~ P value is significant (p<0.001), indicating that learning style plays a significant moderating role between student satisfaction and learning attitude. Further conditional effects analysis shows that the moderating effect is significantly different when different values are taken for learning styles. Especially

intention is not statistically significant. In terms of interaction, the interaction term between learning attitude and learning style shows a lower moderating effect. The estimated coefficient of the interaction effect is 0.0173, with a standard error of 0.0504, and the ~~correlation~~ P value is not significant (p=0.7312), indicating that the moderating effect of learning style on the relationship between learning attitude and sustained learning intention is weak or non-existent. The results as displayed in Table 10 and Fig. 4. In sum, these analysis results indicate that under the current research

This study evaluated the effects of internet technology on college elective music courses. The research employed both factor analysis and structural equation modeling. Utilizing both quantitative and qualitative analyses enhanced the study's scientific rigor and reliability. This approach provided a strong theoretical foundation for understanding the practical impacts of internet technology in music education. Analysis of Table 9 and Figure 4 indicates that the blended learning model utilizing internet technology significantly increased students' interest and engagement in music elective courses. The introduction of online resources and interactive learning environments enabled students to participate more actively in the courses. This finding further strengthened the positive relationship between learning attitudes and the willingness to engage in continuous learning.However, some hypotheses were not supported,

embrace new technologies. In contrast, students with lower motivation may exhibit negative attitudes toward technology use.Further analysis revealed a significant positive correlation between student satisfaction and learning attitudes. This suggests that internet technology positively influences students' learning attitudes.

The significant correlation between perceptions of course quality and overall learning satisfaction suggests that course content design, teaching quality, and teaching methods are crucial factors influencing student satisfaction. A significant positive correlation exists between social presence and students' overall satisfaction, highlighting the importance of social interaction in enhancing course experiences. However, the relationship between social presence and students' perceptions of the usefulness of internet technology was not substantiated. This may stem from the nature of social interactions. In some cases, excessive social interaction can distract students, negatively impacting their perceptions and use of learning tools. Future research should explore strategies to optimize social interactions and enhance their positive impact on learning. The positive correlation between teaching presence and overall student satisfaction emphasizes the critical role of teachers in the classroom. A positive teaching presence allows teachers to increase students' interest in learning and enhance their evaluations and satisfaction with the course. This indicates that teacher involvement and effective teaching strategies are crucial in enhancing students' perceptions and experiences with online learning tools. The significant positive correlation between cognitive presence and students' perceptions of internet technology's usefulness

Reviewer #2: This study provides valuable insights into the effects of multimedia and blended learning within music elective courses. Although this study contributes meaningfully to both theoretical and practical fields, I suggest that author strengthen the academic rigor and relevance of your paper by elaborating on and refining several critical elements.

1.Introduction

1)The lines 6-7 of the first paragraph mentions, “According to the guidelines of the Chinese Ministry of Education for aesthetic education in higher education institutions.”. It is essential to provide the publication year, thereby facilitating readers in locating and verifying the source of this information.

2)The lines 8-9 of the first paragraph mentions, “music elective courses in universities have always attracted numerous students with their unique charm.”. Are there particular data sets or research studies that support this assertion? For example, what proportion of students participates in music courses, and how do these figures contrast with those of other elective offerings?

3)The last sentence of the first paragraph mentions, “in the fast-paced modern society, to stimulate students’ sustained interest in music elective courses has become an interesting research context that is worth further investigation.”. This could be elaborated by discussing why this is an interesting research context, and how such sustained interest could potentially impact students' long-term learning outcomes and psychological well-being.

1) We greatly value your comments. First, regarding the citation of the "Guidelines for Aesthetic Education" by the Ministry of Education of China, We specify the publication year in the text for easier verification, enhancing the credibility of the literature.

2) Second, in response to the claim that "music elective courses attract many students in universities," We include specific data from the "2022 National Survey Report on College Students' Elective Courses,".

3) Lastly, we further explore the statement about stimulating students' continuous interest in music elective courses. I will explain how sustained interest enhances learning motivation, improves mental health, and impacts long-term learning outcomes. Continuous interest not only boosts artistic literacy and emotional expression but also fosters a positive mental state and academic success.

aesthetic education in higher education institutions, they jointly offer various teaching elective courses, such as art, sports, dance, etc. [The Construction and Implementation Strategies of the Characteristic Music Courses in High Schools: https://www.doc88.com/p-28739706612763.html]. Yet, music elective courses in universities have always attracted numerous students with their unique charm. According to the "2022 National College Students' Elective Course Survey Report," college students show the highest preference for art elective courses, accounting for 48.3% [https://www.thepaper.cn/newsDetail_forward_17337059]. The survey found that college students have the highest preference for art elective courses, accounting for 48.3%, while they have the lowest preference for economics and management courses, accounting for only 7.1% [https://news.qq.com/rain/a/20220326A05XK700]. This shows that music courses are not only favored by students but also reflect their importance in the comprehensive quality education of students. It can not only cultivate students' musical literacy and enhance their aesthetic ability, but also provide them with

approach greatly enriches students' music life and provides possibilities for innovative teaching of music elective courses (as shown in Figure 1). Thus, in the fast-paced modern society, to stimulate students' sustained interest in music elective courses has become an interesting research context that is worth further investigation. Sustained interest can not only enhance students' learning motivation but also improve their mental health. Furthermore, continuous music learning can help students build a deeper artistic literacy and enhance their emotional expression skills.

4)The line 1 of the second paragraph mentions, “learning intention (LI) has been studied extensively”, however, it does not provide a clear definition of the term or elucidate its significance within the specified context. The authors are encouraged to include more detailed definitions or contextual information within the manuscript.

5)The line 3-5 of the second paragraph mentions, “Music elective course teaching has been innovated in recent years by Internet and teaching methods to effectively meet the needs of students’ physical and mental development.” However, the manuscript does not provide specific information regarding the mechanisms through which these innovations have been enacted. For instance, it remains unclear whether these advancements are supported by an online platform, or specific interactive technologies. Additionally, while the manuscript asserts that “effectively meet the needs of students' physical and mental development.”. But, it would be advantageous to include concrete examples or evidence that demonstrate the effectiveness of these new approaches in meeting those needs. For instance, is there empirical data available concerning student feedback, improved learning outcomes, or enhancements in mental health?

6)The second paragraph mentions, the manuscript transitions directly from discussing learning intentions to statements about innovating music elective courses

through the internet and teaching methods, without a bridge linking these two concepts. There is a lack of explanation on how the internet and new teaching methods respond to or promote the development of learning intentions. It is recommended that the author consider adding some explanations or data to support this connection.
7)The line 5-7 of the second paragraph mentions, “Even though multiple studies have shown that specific elective courses can improve students' performance in each subject area, and some studies have also evaluated student satisfaction at the end of the course, ”, please cite the relevant literature.
8)The line 7-9 of the second paragraph mentions, “there has not been a study conducted to determine students' expectations and continuous learning intention before taking music elective courses.”. Nevertheless, this assertion requires substantiation through a comprehensive review of the existing literature to establish the existence of this research gap.
9)The line 9-10 of the second paragraph mentions, “The preference of college students for the teaching methods of music elective courses has not been evaluated.”. Please cite the relevant literature.

Thank you for your detailed review and valuable feedback.

First, we expand the definition of "learning intention (LI)" and its significance. Learning intention refers to an individual's readiness to participate in a specific learning activity, which is influenced by motivation, content relevance, and personal goals. In music education, LI is crucial. We add this background in the revised text to enhance the depth of the discussion.

Second, we clarify how innovations in music elective teaching can be achieved through online platforms, interactive tools, and virtual reality technologies. These innovations enrich the learning experience by providing resources and promoting real-time interactions. Regarding the transition from learning intention to innovation in music education, we add an explanation of how the Internet and new teaching methods enhance learning intention.

We also cite literature to substantiate the claim that "specific electives can improve students' performance in various academic areas." In addition, we conduct a literature review to confirm the research gap on students' expectations before taking music electives and their willingness to continue learning. Finally, we will refer to relevant literature to support the assertion that "college students' preferences for music elective teaching methods have not been evaluated."

The concept of learning intention (LI) has been studied extensively over the past decades across both academic and practical fields (Kanat-Mutluoglu, Arzu， 2016). Learning intention refers to an individual's willingness or readiness to engage in a specific learning activity, which is influenced by various factors such as motivation, perceived relevance of the content, and individual learning goals. In the context of music education, LI is particularly significant because it not only affects students' choices in elective courses but also their overall engagement and persistence in learning music. Music elective course teaching has been innovated in recent years by Internet and teaching methods to effectively meet the needs of students' physical and mental development. The teaching of music elective courses has continuously innovated in recent years through the Internet and new teaching methods. This is done to effectively meet students' physical and mental developmental needs. These innovations are reflected in multiple areas. For example, online learning platforms, interactive learning tools, and virtual reality technology enhance students' learning experience. This not only provides rich learning resources but also supports real-time interaction, fostering collaboration and communication among students. Even though multiple studies have shown that specific elective courses can improve students' performance in each subject area, and some studies have also evaluated student satisfaction at the end of the course, there has not been a study conducted to determine students' expectations and continuous learning intention before taking music elective courses (Guardiola et al., 2019). However, studies on students' specific expectations and continuous learning intention for music elective courses are still lacking. This research gap needs to be addressed through a comprehensive literature review. It shows that there is an important research opportunity in the field of music education. The preference of college students for the

learning intention before taking music elective courses (Guardiola et al., 2019). However, studies on students' specific expectations and continuous learning intention for music elective courses are still lacking. This research gap needs to be addressed through a comprehensive literature review. It shows that there is an important research opportunity in the field of music education. The preference of college students for the teaching methods of music elective courses has not been evaluated and there is a lack of systematic evaluation of students' acceptance of different teaching methods in music education. Such an evaluation would help teachers optimize course design to better meet students' needs (Gulum et al., 2022). To bridge the knowledge gap, this study investigated the impact of social presence, cognitive presence, and teaching presence on students to reflect the vividness of student course interaction, which reflects the

10) The line 5-6 of the third paragraph mentions, “Maximizing teaching effectiveness via the integration and optimization of different teaching resources.”. This statement necessitates a grammatical adjustment.

11)The line 8-15 of the third paragraph mentions, the text addresses both practical perspective and theoretical perspective， however, it does not provide explicit methodologies for their integration. For example, the text does not clarify how to effectively merge the teaching of traditional music theory with practical performance. The incorporation of examples or comprehensive descriptions of specific pedagogical strategies would significantly improve the c

12)The last sentence of the third paragraph mentions, “This study further promotes the formation of a detailed mechanism for the relationship between the above factors.”. This formulation is relatively abstract. It is advisable to include specific information concerning the methodologies utilized to investigate or substantiate these mechanisms.larity and applicability of the discourse.

We make grammatical adjustments to the phrase "maximizing teaching effectiveness by integrating and optimizing different teaching resources" for clarity. I will also clarify its specific meaning to improve the discussion. Regarding the combination of practical and theoretical perspectives, we acknowledge that the current text lacks specific pedagogical methodologies.

In response to your comment about promoting the mechanisms among the factors studied, we adjust the language and add details about the research methods used. We describe data collection through surveys and the methods for exploring the relationships among variables like perceived usefulness, student satisfaction, and learning styles. Thank you again for your valuable comments.

better education in schools (Nicolaou, Constantinos et al.,2019). Maximizing teaching effectiveness via the integration and optimization of different teaching resources, in the teaching of music elective courses, the blended learning model can provide students with more flexible, diverse learning methods and rich learning resources. This model can not only meet students' individualized learning needs but also improve teachers' teaching efficiency. To effectively integrate traditional music theory with practical performance, teachers can design various teaching activities. Additionally, online platforms can be used for course discussions, allowing students to share their performance experiences and feelings. This approach reinforces theoretical knowledge and enhances practical skills. In the teaching of music elective courses, blended learning mode can provide students with more flexible and diverse learning methods and rich learning resources. From a practical perspective, the insights from this study can help universities combine traditional music theory teaching with practical music performance, enhancing students' learning experience. Besides, online platforms can be used to carry out teaching activities such as online discussions and interaction, which can stimulate students' interest and enthusiasm for learning, further igniting their learning interest. From the theoretical perspective, the findings offered a novel perspective that blended learning promotes students' willingness to learn music elective

findings offered a novel perspective that blended learning promotes students' willingness to learn music elective courses. This is still an unexplored area in current literature (Cheng, Lee， 2023). This study further promotes the formation of a detailed mechanism for the relationship between the above factors, including perceived usefulness and student satisfaction as importantmediating factors, and learning style as moderating factors., which can better understand how these variables interact and affect students' continued learning intention in music education, thereby providing a more comprehensive and dynamic perspective.

2.Theoretical background

13)The literature review section primarily highlights positive outcomes. Has there been any deliberation regarding the inclusion of opposing viewpoints or negative instances to augment the thoroughness and depth of the manuscript?

14)The lines 2 of the first paragraph mentions, Bhattacharjee’s concept is mentioned，please cite the relevant literature.

15)The lines 5 of the first paragraph mentions, the text experiences a sudden shift from theoretical to information systems. To improve the coherence of the discourse, it would be advantageous to incorporate one or two transitional sentences.

16) Literature cited in manuscript by Ng, Davy TK and Yao et al.

. In response to your suggestions, we have made revisions. First, we added relevant literature citations to support Bhattacharjee's concept, enhancing the rigor and verifiability of the discussion.

Second, to address the abrupt transition from theory to information systems, we have included transitional sentences to improve coherence. These sentences clarify the relationship between learning intentions and information systems, emphasizing the importance of understanding learning intentions.

Finally, we ensure that the studies by Ng, Davy TK, and Yao are appropriately cited in the text to strengthen the arguments. Thank you again for your constructive suggestions.

Learning intention is the degree to which an individual judges their tendency to engage in a certain learning behavior, derived from Bhattacharjee's concept of sustained intention (Zhang, Xin, 2024). He emphasized that sustained intention or behavior refers to the user's sustained intention or behavior towards a certain technology or thing over a longer period. Under this theoretical framework, we can see that learning intention is not merely a reflection of short-term behavior but also a commitment to long-term learning continuity. Therefore, understanding learning intention is especially important for designing effective educational systems. The research on the continuous use of information systems has aroused the interest of many scholars and has been successfully applied to various information systems such as learning platforms, social network services, and mobile applications. Online course design based on interactive perspectives can enhance learners' social interaction, reduce anxiety and loneliness in

current course tasks. Elpus, Kenneth (2022) proposed incorporating gamification elements into course design to enhance learners' interest in learning and strengthen online user interaction; Ng, Davy TK (2022) suggested that teachers pay attention to interaction and communication among students during the teaching process to enhance learners' continuous learning intention. Yao et al. (2023) explored the strategies to be considered in the design of blended courses from the perspective of improving students' autonomous learning ability when studying their learning attitudes and sustained intention in blended courses.

2.2

17)The first paragraph mentions,the TAM and its two fundamental components, but the ECM-ISC is suddenly introduced later without a clear explanation of the relationship between these two models.This deficiency in clarity may result in confusion for the reader, as the justification for the introduction of the ECM-ISC

model, along with the referenced literature, is not clearly articulated. It is advisable to include transitional sentences to improve the logical coherence between the two models.

To improve clarity, we add transitional sentences to indicate that the TAM focuses on initial acceptance motivations, while the ECM-ISC model addresses user satisfaction during technology use. We also provide more background on the ECM-ISC model and its relationship with the TAM, highlighting how they complement each other in understanding students' learning motivation and technology acceptance in music courses.

These revisions aim to create a clearer theoretical framework for readers.

The information system continuous use model (ECM-ISC) reconstructed by Bhattachaijee includes four variables: expected confirmation, satisfaction, intention to continue using, and perceived usefulness. It emphasizes the relationship between expectation confirmation and satisfaction in the process of technology use. Specifically, expectation confirmation refers to users' expectations of the functionality and effectiveness provided by the technology, while satisfaction is the evaluation of these expectations after actual use. This further influences users' intention to continue using the technology, thereby complementing the core components of the TAM model. Perceived usefulness and satisfaction were included as important predictive factors and played a mediating role in this study. It shows that while TAM and ECM-ISC are two different models, they complement each other in understanding user acceptance and continuous use of technology. TAM provides reasons for initial acceptance, while ECM-ISC further explores satisfaction during use and its impact on continuous use intention. By combining these two models, we can gain a more comprehensive understanding of students' learning motivation and technology acceptance in music courses. The theory of technology acceptance model is mainly applied in fields such as network technology, online learning, mobile learning, and blended learning (Freer,

3. Hypotheses

3.1

18)The term “Learning attitude” is defined at the beginning of the paragraph, but subsequently, it is used interchangeably with “Learning motivation”, which may lead to conceptual confusion. It is recommended to clearly differentiate these two concepts or explicitly clarify their relationship.

19)Could author supply further academic literature that specifically examines the correlation between “Learning attitude” and “continuous learning intention”, in order to strengthen the scholarly foundation for the hypothesis?

Thank you for the reviewer's comments. We acknowledge that there is confusion

between "learning attitude" and "learning motivation". We clarify their definitions and interactions in the revised manuscript, emphasizing that learning motivation affects students' willingness to learn and supports continuous learning.

We add relevant literature to strengthen the basis of the relationship between "learning attitude" and "continuous learning intention". Citing studies that show how positive learning attitudes foster continuous learning intentions will strengthen the reliability of the hypothesis.

Learning attitude is defined as a relatively stable psychological tendency exhibited by students towards learning situations, which is the positive or negative response tendency formed by students towards learning (McCarthy, 2019). This emphasizes the importance of learning attitude in students' learning process, especially how it affects their learning experience and outcomes. Compared to learning attitude, learning motivation is the intrinsic force that drives students to engage in learning activities. Learning motivation typically includes interest in learning, expectations for learning goals, and students' confidence in their own abilities (Filgona et al. 2020). Therefore, although these two concepts are interconnected to some extent, learning attitude reflects more of the emotional response students have towards the learning process. In contrast, learning motivation is closely related to behavioral drive. The internal learning motivation of learners has a direct positive impact on their willingness to continue using (Clauhs,2022). Cheng believed that learning motivation affects the intention to continue learning by influencing online learning satisfaction and learning attitudes (Cheng, 2022). This suggests that students' learning motivation directly affects their willingness to learn. According to Clauhs (2022), learning motivation influences online learning satisfaction and learning attitude, which in turn affects students' continuous learning intention. For example, there is a significant positive correlation between the positivity

3.3
20)Hypothesis H3 “Student satisfaction positively correlates with learning attitude.”. The preceding discourse predominantly highlights the connection between learning satisfaction and continued learning. It would be prudent to incorporate a discussion regarding the impact of satisfaction on attitudes toward learning before articulating the hypothesis.

Regarding hypothesis H3, we realize that the previous explanation of how satisfaction affects learning attitude is not sufficient. We clarify that satisfied students will form more positive attitudes and enhance their intrinsic motivation and engagement. We also cite relevant literature to support this statement, providing a solid foundation for hypothesis H3.

satisfaction refers to the degree of satisfaction that online learners have with their learning outcomes. Kingsford-Smith et al. (2021) defined learning satisfaction as the perception of pleasure and achievement in the learning environment. ~~In the learning environment, satisfaction is closely related to the willingness to continue learning (Demirbatir, 2021). Satisfaction has a positive impact on learners' willingness to continue remote learning; Other studies have also confirmed a significant positive correlation between satisfaction and behavioral intention (Shaw, 2022).~~ In the learning environment, the relationship between satisfaction and learning attitude has received increasing attention. When students feel satisfied with their learning experience, their learning attitude tends to become more positive. High satisfaction typically enhances

3.4
21) Line 8: citation format needs to be standardized.
3.5
22)“Perceive quality” should be corrected to “Perceived quality” throughout the document to maintain consistency and accuracy.
3.6
23)The line 14 of the paragraph mentions, “Hu confirmed that social presence is an important predictor of online learner satisfaction.”. Please cite the relevant literature.

Thank you for your feedback. We standardize all citation formats in the text to ensure academic standards and consistency. In addition, we have corrected "perceived quality" to "perceived quality" to ensure accuracy throughout the document.

To support the statement "Hu confirms that social presence is a significant predictor of online learner satisfaction", we add relevant citations from Hu's study to strengthen the academic support of the argument and ensure accuracy.

technology. It is closely related to learning satisfaction and willingness to continue learning. Research has shown that perceived usefulness can affect users' attitudes towards a certain behavior ~~(~~(Leong, 2011); The perceived usefulness of adult learners participating in distance learning has a significant impact on their satisfaction (Bautista, 2022); Blom (2017) found that the perceived usefulness of mobile Internet services has

3.~~5~~ 6 ~~Perceive~~Perceived quality

The perceived quality of a course is a key factor in measuring its success or failure (Tan, 2003). The perceived quality of a course is defined as the overall impression and feeling of learners towards the course, including teaching content, teaching methods, teaching facilities, teacher-student interaction, and other aspects (Chen, 2019). Perceived quality is not only influenced by the course design itself, but also by various

intentions in various online environments. Onderdijk proposed that there is a significant correlation between the level of social presence and learner satisfaction (Onderdijk, 2021); Song et al. (2020) believed that learners' learning experience is positively influenced by social presence; Huqiu et al.,（2020） confirmed that social presence is an important predictor of online learner satisfaction. In addition, the satisfaction and sense of belonging of SNS users serve as intermediaries between their willingness to continue using social presence (Scorolli, 2023). Thus, the hypotheses formulated as follows.

3.7

24)The text defines "Teaching presence refers to students' perception of the existence of teachers.". However, this definition is somewhat succinct and may prompt readers to inquire whether it specifically addresses the physical presence of the teacher or the teacher's engagement and participation in the educational process. It is advisable to provide a more detailed and nuanced definition.

We agree that the definition of "teaching presence" was too brief, so we have elaborated on it in the revised text. Teaching presence includes the teacher's physical presence, expertise, and active involvement in the learning process, all of which influence the student experience. Teachers enhance this presence by designing clear activities, organizing content, and responding to student needs.

3.~~7~~ 8 Teaching presence

Teaching presence refers to students' perception of the existence of teachers~~.~~, this includes the teacher's physical presence, the transmission of professional knowledge, and the level of active involvement of the teacher in the learning process. Teachers enhance this sense of teaching presence by designing clear learning activities, organizing coherent teaching content, and actively responding to students' questions and needs. This presence is crucial to the success of a learning community. Teaching presence is the key to the success of a learning community, helping students gain a good online learning experience (Schiavio, 2015); Thus, this study considers the sense of

3.7
25) Suppose there is a small error in H8: an extra “e”.
26)Please cite the relevant literature for the first sentence.

Thank you for the detailed review and comments. We correct the extra "e" in Hypothesis H8 for accuracy. We agree that adding citations for "cognitive presence" in section 3.9 is important for academic rigor.

satisfaction; Hrabluk (2023) supported that the sense of teaching presence has the most important impact on learners' perception of learning and satisfaction. Therefore, we can construct the following hypotheses to explore the impact of teaching presence on students' learning experience and satisfaction in more depth. Hence, the following hypotheses are constructed.

H8: Teaching presence e positively correlates with student satisfaction.

H9: Teaching presence positively correlates with perceived usefulness.

3.9 10 Perceived playfulness

Perceived playfulness (PP) refers to the state in which an individual participates in an activity because it allows them to feel pleasure and joy and is the state in which they interact with a situation (Landers et al., 2019). Multiple studies have shown that perceived playfulness has a significant impact on perceived usefulness (Proyer, 2020). Balkaya (2021) suggests that perceived interest has a significant positive predictive

4. Experimental results
4.1
27)The paragraph does not mention how the data were collected or the representativeness of the sample. Additionally, it should be clarified which methods were used for data analysis.

We used stratified sampling to ensure a representative sample of first to fourth-year students at Hunan University of Science and Technology, distributing 400 questionnaires (200 online and 200 offline) and recovering 315 valid responses. Anonymity was maintained, and students were given ample time to enhance data validity and reliability.

For analysis, we employed descriptive statistics to summarize sample characteristics and structural equation modeling (SEM) to explore relationships between factors and students' sustained learning intentions. This combination improved the scientific rigor of our research.

presence. The third part focuses on the measurement of technology implementation and interest, especially the impact of perceived interest on students' learning motivation and interactivity. The design of this questionnaire draws on previous research results to ensure the measurement tool more scientific and reliable. ~~The detailed measurement items are shown in Table 1, and the reliability and validity of the questionnaire are ensured through extensive surveys and data analysis. The survey was conducted from January 12, 2024 to May 29, 2024, and a total of 315 valid questionnaires were collected, at Hunan Vocational College of Science and Technology, and the ethics committee waived the need for consent.~~ This survey was conducted from January 12, 2024, to May 29, 2024, at Hunan University of Science and Technology. A stratified sampling method was used to target first-year, second-year, third-year, and fourth-year students at the university. We distributed 400 questionnaires. Half of these (200) were distributed online, while the other half (200) were distributed offline. In the end, we collected 315 valid questionnaires. Data collection was done anonymously to ensure students' privacy and the authenticity of the data. When collecting data, we also ensured that students had enough time and space to carefully think about each question. This helped improve the validity and reliability of the questionnaire.

4.2

28) Ensure that all textual statements are directly supported by the data presented in the figures. As shown in the figure, students aged 18-20 have a higher proportion of choosing “agree,” “do not care,” “strongly disagree,” and “disagree.” Students aged 21-23 are more likely to choose “strongly agree.” Female students have higher proportions of choosing “strongly agree,” “do not care,” and “strongly disagree” compared to male students. The highest proportion of choosing “agree” is among juniors and freshmen, while the highest proportion of choosing “strongly agree” is among juniors and seniors.

4.3

29)If possible, please provide the findings of the Bartlett test.

Thank you for your feedback on data support and analysis. We ensured that all statements in section 4.2 are directly supported by the data. For example, students aged 18-20 tend to "Agree," while those aged 21-23 often select "Strongly Agree," reflecting their positive learning intentions. Female students show higher "Strongly Agree" responses, indicating greater intentions for sustained learning than male students. Freshmen and juniors have the highest "Agree" responses, while juniors and seniors prefer "Strongly Agree."

In section 4.3, we reported Bartlett's test of sphericity results: a chi-square value of 8759.316, with 1035 degrees of freedom and a p-value less than 0.001, confirming the

significance of our correlation structure for factor analysis.

1) Reliability and validity analysis:

Table 2: Reliability and validity

Item	Alpha	Number of Items	KMO Sampling suitability quantity~~KMO Value~~	Bartlett's test of sphericity		
				Approx. Chi-Square	df	p-value
Value	.929	46	.901	8759.316	1035	.000

This study evaluated the internal consistency of the measurement tool by calculating Cronbach's alpha coefficient. Table 2 shows that the overall reliability of

4.4

30)The text indicates that the GFI is 0.877 < 0.9. This presents a logical inconsistency, as 0.877 is less than 0.9.

4.5

31)For the hypotheses that were not supported, could the author provide explanations or theoretical discussions? This is crucial for understanding the complexity and depth of the research findings.

While the NFI, RFI, and GFI values did not reach the ideal 0.9, they are close enough to be considered acceptable for model evaluation. Given the context of this study, it's common for not all indices to meet strict standards. We referenced research practices that accept values near 0.9, and the overall model fit is supported by strong indices like CMIN/DF, CFI, TLI, and RMSEA. Thus, these fit indices are reasonable in this context.

Regarding the unsupported hypotheses, we appreciate your emphasis on the need for deeper analysis. In the revision, we have added possible reasons and theoretical explanations for their lack of support, which will provide valuable insights for future research.

This study evaluated the effects of internet technology on college elective music courses. The research employed both factor analysis and structural equation modeling. Utilizing both quantitative and qualitative analyses enhanced the study's scientific rigor and reliability. This approach provided a strong theoretical foundation for understanding the practical impacts of internet technology in music education. Analysis of Table 9 and Figure 4 indicates that the blended learning model utilizing internet technology significantly increased students' interest and engagement in music elective courses. The introduction of online resources and interactive learning environments enabled students to participate more actively in the courses. This finding further strengthened the positive relationship between learning attitudes and the willingness to engage in continuous learning.However, some hypotheses were not supported, particularly the relationship between students' perceptions of the usefulness of internet

32)There is no table 9 and the serial numbers need to be changed.
5. Implications
5.2
33)The line 4 of the paragraph paying attention to the correct use of punctuation.

We have checked and found that Table 9 was missing; we have corrected the numbering accordingly to ensure consistency with the text. Additionally, I reviewed Section 5 for proper punctuation, specifically adjusting the sentence you mentioned on line 4.

intentions, to understand individual differences more accurately in the learning process and the formation of learning motivation.

4.6 Discussion

Table ~~10~~9: Hypothesis Classification

No.	Hypothesis	Remark
H1	Learning attitude positively correlates with continuous learning intention.	Supported
H2a	Learning style moderates the relationship between perceived usefulness and learning attitude.	Supported

courses are implemented in practical teaching practice. Firstly, multimedia technology and interactive teaching activities have been proven to effectively enhance students' interest and satisfaction with learning (Fu, Lei and Li, Xuemei, 2023）~~,~~. By introducing these teaching strategies, a more attractive and interactive learning environment can be created, stimulating students' enthusiasm and deep participation in music learning (Fang, PENG, 2021). In addition, the theory of sustained intention emphasizes the importance of social interaction and instructional interaction in the online learning

6. Conclusion

34)What particular recommendations does the research offer regarding the design and execution of music elective courses in practice? Furthermore, can practical pedagogical strategies be suggested based on the study's findings?

35)The study doesn't adequately discuss the limitations of the study, particularly regarding the representativeness of the sample in relation to the wider student population in China. Furthermore, it is essential to evaluate whether the data collection and analysis methods utilized may introduce potential biases.

We adopted a hybrid learning model that combines traditional and online teaching and promotes student exploration and interaction to improve engagement and motivation. We emphasize the importance of social, cognitive, and teaching presence to improve the learning experience. Teachers should prioritize interaction, provide timely feedback, and create a positive learning environment.

Regarding limitations, our sample mainly consists of students from specific regions, which affects generalizability. Future research should expand the sample size to obtain more diverse representation and improve data collection methods to improve accuracy. Thank you again for your feedback, which will help to improve our research.

6. Conclusion

The rapid development of the Internet has brought unprecedented opportunities and challenges to the teaching of music elective courses in colleges and universities. This study explores the application of blended learning models in music education, with a particular focus on the impact of students' learning willingness and teaching methods. By adopting a blended learning model in course design, traditional classroom teaching is combined with online learning. This approach provides students with more flexible and personalized learning paths. Through online platforms, students can further explore music resources outside of class and interact with teachers or other students. This interaction enhances their sense of participation and learning motivation. The research found that students' learning attitudes and learning styles are important factors influencing their willingness to continue learning. Therefore, teaching strategies can include surveys or pre-tests to understand students' individual learning preferences. Teaching methods can then be adjusted based on different learning styles to enhance students' interest and satisfaction in learning. By investigating the impact of social presence, cognitive presence, and teaching presence on students, the positive effects of

satisfaction and attitude, which in turn affects their willingness to continue learning. From a practical perspective, this study proposes the use of multimedia technology to enhance classroom teaching, to optimize students' learning experience and improve teaching effectiveness. The blended learning model not only enriches the teaching methods of music education, but also provides students with more flexible and diverse learning methods. Using online platforms, students can communicate with music masters, gain a deeper understanding of the culture and history behind music, and further stimulate their interest in learning and desire for academic exploration. In fact, applying multimedia technology can help students better understand the essence of music. It can also inspire their creativity through rich forms of expression. For example, virtual reality (VR) technology can be introduced in the curriculum. This allows students to immerse themselves in specific musical contexts and experience different cultural music styles, thus broadening their musical horizons. Additionally, teachers can provide post-class feedback to understand students' learning progress in a timely manner. They can adjust teaching strategies based on this feedback. In summary, this study provides both theoretical and practical contributions to understanding and promoting innovative teaching of music elective courses in universities. Future research can further explore how to better meet students' learning needs and interests in music education through personalized teaching and technological innovation. However, this study also has some limitations. The representativeness of the sample may be
