# Supplementary material for: How do multimedia and blended learning enhance music elective courses? examining the roles of learning attitudes, styles, and teaching presence
Source: PLoS One. 2025 Jun 25;20(6):e0326037. doi: 10.1371/journal.pone.0326037 (PMC12192290; doi:10.1371/journal.pone.0326037)
Supplement: Attachment — Submitted filename: Response to Reviewers-20.Feb.docx [file pone.0326037.s006.docx]

Dear Editor and Reviewers,

We sincerely appreciate the time and effort you have dedicated to reviewing our manuscript titled “How Do Multimedia and Blended Learning Enhance Music Elective Courses? Examining the Roles of Learning Attitudes, Styles, and Teaching Presence”. This study aims to explore the possibility of using Internet resources to enhance the educational effect of music elective courses in colleges and universities. The research results are of great significance for optimizing the design and teaching methods of music elective courses, providing theoretical support and empirical basis for promoting innovation in music education. We are grateful for the constructive feedback provided by Reviewer #1 and Reviewer #3, which has helped us improve the quality of our work. Below, we address the reviewer’s comments point by point and outline the revisions we have made to the manuscript. We believe that these revisions have significantly strengthened the manuscript and addressed the concerns raised by the reviewer. We are confident that the revised version meets the high standards of PLOS ONE and hope that it is now suitable for publication.

Thank you once again for your valuable feedback. We look forward to hearing from you regarding the next steps in the review process.

Sincerely,

Pingbo Tang

Hunan Vocational College of Science and Technology

**Response to Reviewers' Comments：**

Reviewer #1:

1) Remove NFI, GFI, and RFI results from Table 4. Moreover, the comments related to these GoF tests should be also removed.

Thank you for your valuable feedback on our manuscript. We have made corrections regarding the expression of NFI, GFI, and RFI values.

Reviewer #1:

2) How to determine sample size in your study? As i understood, the numbers of participants was 315. Is this meet your predetermined sample size?

We appreciate your feedback. You have inquired about the determination of our sample size and whether the number of participants (315) meets our predetermined sample size. In our study, the sample size was determined based on a power analysis conducted prior to data collection. The power analysis indicated that a sample size of 315 would provide sufficient statistical power to detect significant effects, given the expected effect sizes and the complexity of our structural equation model (SEM). Therefore, we can confirm that the number of participants (315) does meet our predetermined sample size. To clarify this point, we have added a new paragraph in the Methodology section of the manuscript, explaining the process of sample size determination and providing the rationale behind our choice. This will help readers understand how we arrived at the sample size and ensure transparency in our research methods.

Reviewer #1:

3) In SEM applications, both exploratory factor analysis (EFA) and SEM are not performed on the same data. Because a second data collection process is required to verify the exploratory design. And for this purpose, a CFA should be used to test the measurement model. As far as I understand, in your study, both EFA and SEM were performed with the same data. If there is a scientific basis to this, please indicate it in the study by citing the relevant scientific sources.

We recognize that EFA (Exploratory Factor Analysis) and SEM (Structural Equation Modeling) are often based on different datasets, with EFA aimed at discovering potential factor structures and CFA (Confirmatory Factor Analysis) used to validate predetermined structures. Traditionally, both use independent datasets. Given the large sample size and high data quality of this study, and the suitability for factor analysis confirmed by KMO (0.901) and Bartlett's test (p<0.001), we have decided to use the same dataset for EFA and SEM. CFA successfully validated the factor structure obtained by EFA, and SEM path analysis also passed the fitting and significance tests, enhancing the effectiveness of the model. We refer to Kyriazos' (2018) study, which suggests that when the data is of high quality and the sample is sufficient, EFA, CFA, and SEM can share datasets. EFA can provide a reliable basis for subsequent analysis without the need for additional data collection. Meanwhile, we ensured the simplicity of the factor structure to reduce the risk of overfitting. The validation of CFA has laid a solid foundation for SEM analysis. Despite using the same dataset, we ensured the stability, credibility, and rationality of the model through multiple validations. The study has elaborated on this method in detail and cited literature such as Kyriazos (2018) as theoretical support.

Reviewer #3:

1. There are several points of inconsistency, namely:

a. Reference formatting. Style changes between sections but never in Vancouver style as stipulated in the PLOS submission guidelines. In-text citations seem to be missing in the bibliography and/or vice-versa, for example Guardiola et al., 2019 and Rodríguez et al., 2019, respectively, unless this is a typographical error? In any case, this should be checked carefully.

b. Plot and table formatting. These change throughout the document. In Figure 3 especially the text is quite small and hard to read.

We acknowledge the inconsistencies in the reference formatting and have now standardized all references to follow the Vancouver style as stipulated in the PLOS submission guidelines. We have carefully reviewed and corrected any deviations from this style throughout the manuscript. We have addressed the inconsistency in plot and table formatting by ensuring a uniform style throughout the document. For Figure 3, we have increased the font size of the text to improve readability. Additionally, we have reviewed all other figures and tables to ensure they meet the required standards for clarity and presentation.

Reviewer #3:

c. Statistical reporting. Sometimes significance is referred to with p< statements, others precise p values are given. I don’t think using asterisks in text is necessary.

d. Writing quality. Some sections read quite clearly whereas others are quite difficult with long sentences and often statements that are rephrased and repeated. For example, in the introduction: “According to the "2022 National College Students' Elective Course Survey Report," college students show the highest preference for art elective courses, accounting for 48.3% [https://www.thepaper.cn/newsDetail_forward_17337059]. The survey found that college students have the highest preference for art elective courses, accounting for 48.3%, while they have the lowest preference for economics and management courses, accounting for only 7.1% [https://news.qq.com/rain/a/20220326A05XK700].” The writing throughout could be made more concise to help the reader follow the article. Proofreading should treat the article as one whole not in segmented parts. Further, check the appropriate referencing format for citing websites.

We agree that maintaining consistency in statistical reporting is crucial. We have revised the manuscript to ensure that significance is consistently referred to either with p< statements or precise p values, as appropriate. We have removed the use of asterisks in the text and standardized the reporting method throughout the manuscript. We have taken your comments on writing quality to heart and have revised the manuscript to enhance clarity and conciseness. We have rewritten some sections, particularly in the introduction, to eliminate repetition and reduce the length of complex sentences. For example, the repeated information in the introduction about college students' preferences for art elective courses has been consolidated into a single, concise statement. Citing Websites: We have reviewed the appropriate referencing format for citing websites and have corrected any instances where the format was incorrect.

Reviewer #3:

2. Consider how the article is structured. There is no explicit methods section; details are given in the Experimental Results section, but it would help the flow of the article and for the reader to understand what has been done and how it was analyzed (see below) if there were a separate detailed methods (and analysis) description before reporting the results. It is a bit difficult to follow as it currently is.

We appreciate your helpful review and comments. We understand the importance of a clear and separate methods section for the reader's understanding. We have rearranged the manuscript to include a detailed methods section before the Experimental Results section. This section now provides a comprehensive description of the methods used and the analysis conducted, making it easier for readers to follow the research process.

Reviewer #3:

3. Throughout the article you make statements that are unsubstantiated and should be backed up with references, for example in the Introduction “It can not only cultivate students' musical literacy and enhance their aesthetic ability, but also provide them with a channel to express emotions and release stress” / “Sustained interest can not only enhance students' learning motivation but also improve their mental health. Furthermore, continuous music learning can help students build a deeper artistic literacy and enhance their emotional expression skills”; in the Experimental Results section “This may be because female students are more susceptible to emotional influences from music and are more inclined to enrich their spiritual lives through music elective courses”. What is your basis of these statements? Any assertions or declarative statements should be backed up with legitimate academic sources. If there are none, the authors should clearly state where this is an expression of opinion.

Thank you for your careful consideration and valuable suggestions. We acknowledge the importance of supporting our claims with empirical evidence. Therefore, we consulted a lot of relevant literature and cited them to enhance the readability of the study. Even the entire study has been optimized and modified by us.

Reviewer #3:

1. Experimental results

a. The questionnaire items could be more clearly signposted. Table 1 tells you the sources but not the actual questions, and there is no direction in the article to guide the reader to the supplementary material. Additionally, it would be interesting to see the loading coefficients for each item from the PCA. This could be included in a summary table which, if not added to the main article, should include a clear note about where it can be found. This would help not only for the reader to understand the details of the study but also increase the thoroughness of the results presentation. Presumably the ten components derived from the PCA reflect the ten questionnaire categories/constructs? This is also not explicitly stated. If it is not the case, it would be interesting to know and discuss. Did the PCA produce a simple solution? Or was there any overlap of some items across components?

b. Check for language accuracy and/or typos. For example, the second sentence of the Distribution of Elective Courses section says, ‘Based on the results in Figure 2...’, but Figure 2 depicts a model and does not show any results.

Thank you for your valuable suggestions. First, regarding the labelling of questionnaire items and guidance on supplementary materials, we have added a detailed questionnaire information table in the article, listing the specific question items under each construct and placing the table before the references. In addition, we have clearly stated the access path for supplementary materials in the article, making it convenient for readers to obtain complete questionnaires and other related data. In response to your question about the load factor, we have compiled the rotated component matrix and the load factor for each item into a table and presented it in the main text. This table helps readers understand the classification of each item in PCA. For the question of whether the ten factors obtained by PCA reflect the ten questionnaire categories, we further analysed the matching between factor structure and questionnaire construct. The results showed that the factors extracted by PCA were basically consistent with the constructed questionnaire, and each factor effectively represented the corresponding measurement item. Moreover, we also checked the accuracy of the language. These modifications have improved the transparency of the paper and the reliability of the research results.

Reviewer #3:

1. Experimental results

c. I am not sure I agree with your interpretations of Figure 3. Firstly, there is quite an imbalance of numbers in some of the groupings, especially age. This should at the very least be acknowledged as a limitation when making any group-based comparisons. Also, is the title accurate? ‘Distribution of elective courses’, but the labels state ‘continuous learning intention’. You state that the 21-23 group were highest in the ‘agree’ and ‘strongly agree’ categories yet that’s only the case in the latter; the 18-20 group have a much higher count in ‘agree’. Unless you are referring to differences within that age group only, in which case that should be made clearer. You also say that students 18-20 have a higher proportion in the disagree categories; that appears to be true in terms of magnitude, but this plot isn’t showing the data proportionally, and as previously stated there appears to be an imbalance of numbers between those groupings. You also say that students aged 24-26 mainly focused on the ‘don’t care’ option, but that’s not what the plot shows either. Think carefully about how you are presenting your sample. You should explicitly state (or perhaps present in a table) a breakdown of your sample in terms of relevant demographics; it is not easy to glean those details from the figure presented. Demographic information should be included as a reporting necessity but be careful of what comparisons you make (if any) if your groupings are heavily skewed, or any suggestions of their implications. I would avoid making statements about ‘significant differences’ if you have not performed any statistical tests.

We acknowledge the imbalance in the number of participants across some age groups and agree that this is a limitation. To address this concern, we have revised the text to explicitly mention this limitation when discussing group-based comparisons. We have also taken your advice to avoid making statements about 'significant differences' without performing statistical tests. We have removed any such statements from the text. We have re-organized this section of the manuscript to address your concerns and improve the clarity and accuracy of our interpretations.

Reviewer #3:

1. Experimental results

d. Consider the structuring of this section. It may help the reader if you begin with a description and explanation of all the planned statistical analysis before presenting the results of each, in a logical order. Tables should come after they are referred to in the text.

e. A statement already commented on in the previous review still needs addressing: “the GFI is 0.877, which also exceeds the standard value of 0.9”. I believe you are suggesting that you consider values in the region of 0.9 to be considered acceptable, but this has not been clearly articulated and the sentence is, nonetheless, incorrect.

We have reorganized the structure of the study. Moreover, we cite Huang&Lee's (2013) research to support the validity of GFI values, indicating that they meet the basic requirements for model validation. For NFI and RFI, we have retained the results and restated their roles to avoid misleading readers. We have adjusted the discussion format to emphasize the applicability of these indicators in this study and their contribution to the evaluation of model fit.

Reviewer #3:

2. Conclusion

a. I would consider your interpretation of the results. You state, “Analysis of Table 9 and Figure 4 indicates that the blended learning model utilizing internet technology significantly increased students' interest and engagement in music elective courses”, but to show an increase you would have to have compared between different forms of learning, or over time, which you have not. What your results appear to show, if I have understood correctly, is associations between different themes and metrics and students’ attitudes to learning. This is still a valuable insight but consider your terminology carefully. Similarly, “Through comprehensive analysis of quantitative and

qualitative data, this study confirmed the effectiveness of internet technology in enhancing students' learning interest, increasing satisfaction, and promoting continuous learning”. The language used suggests a comparative assessment (i.e., internet technology enhanced/increased/promoted learning compared to something else), which is misleading.

Thank you very much for your insightful comments and suggestions. In response to your concern regarding the terminology used in interpreting the results, we acknowledge that our initial statements could be misleading due to the lack of a comparative assessment between different forms of learning or over time. To address this, we have revised the conclusion.
