# Supplementary material for: How do multimedia and blended learning enhance music elective courses? examining the roles of learning attitudes, styles, and teaching presence
Source: PLoS One. 2025 Jun 25;20(6):e0326037. doi: 10.1371/journal.pone.0326037 (PMC12192290; doi:10.1371/journal.pone.0326037)
Supplement: Attachment — Submitted filename: Response to Reviewers.docx [file pone.0326037.s007.docx]

Dear Reviewers,

We are deeply grateful for your thorough and constructive feedback on our manuscript, "How Do Multimedia and Blended Learning Enhance Music Elective Courses? Examining the Roles of Learning Attitudes, Styles, and Teaching Presence". Your insightful comments have been invaluable in helping us improve the quality and clarity of our work. We sincerely appreciate the time and effort you have dedicated to reviewing our paper, and we have carefully addressed each of your concerns in this revision. Your thoughtful comments have helped us significantly improve the quality of our work. We have carefully addressed each of your concerns and believe the revised manuscript is much stronger as a result. If you have any additional suggestions, we would be happy to consider them. Below, we provide a detailed response to your specific comments.

Thank you for your valuable contribution to our work.

Sincerely

Pingbo Tang

Hunan Vocational College of Science and Technology

**Response to comments**

Reviewer #3:

The structure of the results section is still a bit meandering and confusing. I suggested a formal Analysis section; this would outline what procedures were followed and include any necessary justifications. For example, the explanation for applying EFA, CFA, and SEM on the same dataset, explained in section 4.5, would go there, before you report the results of these analyses. A strict Analysis section may not be necessary, but just as a way of thinking about how things are presented. At the moment, it is a bit odd to report half of the results, then give some explanation of your analysis method, before providing the rest of the results.

We sincerely appreciate your suggestions for improving the organization of the results section. We have reorganized the entire results section to follow logical order:

4. Results

4.1 Source of Questionnaire Questions

4.2 Sample

4.3 EFA Results

4.4 CFA Results

4.5 SEM Results

4.6 Hypothesis Testing

We believe that these changes have greatly improved the process and readability of this section.

Reviewer #3:

Some of the citations are a bit confusing. For example, reference to Song (2023) in the introduction, first paragraph, second to last sentence – the sentence makes a strong statement about music education; however, the cited article does not discuss music. Is this a speculative statement? Do you mean ‘music leaning activities may facilitate the development...’?

We sincerely appreciate your meticulous reading and constructive feedback regarding citation accuracy. Concerning the reference to Song (2023), we removed this citation entirely from the introduction.

Reviewer #3:

Check specifics of Vancouver referencing – in-text citations should be numbered.

We greatly appreciate your careful attention to the reference details. We have implemented the following comprehensive revisions regarding the requirements for Vancouver style.

Reviewer #3:

Check statistical reporting standards, for example “χ2(df) = ” instead of “Chi Squared = / df = ”. Additionally, although you’ve removed the asterixis when reporting significance the sentences now look a bit odd, for example “the path coefficient of perceived usefulness on perceived playfulness is 0.198, with a significance level (p<0.001)”, why not “the path coefficient [...] is 0.198, which was significant (p < .001)”, or “the path coefficient [...] was significant (p < .001) at 0.198,”? (p in italics and removing the 0 before the decimal is commonly done)

We are deeply grateful for your meticulous guidance on statistical reporting standards. We have implemented comprehensive revisions throughout the manuscript: Chi-square Test Formatting and Standardized all reports to "χ²(df) = value" format. Removal of leading zero (e.g., "p = .015").

Reviewer #3:

Table formatting could still be improved, for example a description for Table 4 would be helpful – presumably the acronyms listed as each variable refer to the different measurement items, but some clarification/explicit statement and perhaps a reminder of what they are would be helpful. Table 6 (and others) – words breaking across lines.

We sincerely appreciate your valuable suggestions for improving the display of dining tables. We have adjusted the format of all tables and added the corresponding measurement items for the abbreviations listed in each variable.
